# Canine invasive mammary carcinomas as models of human breast cancer. Part 2: immunophenotypes and prognostic significance

**DOI:** 10.1007/s10549-017-4542-8

**Published:** 2017-10-23

**Authors:** Jérôme Abadie, Frédérique Nguyen, Delphine Loussouarn, Laura Peña, Adelina Gama, Natascha Rieder, Anton Belousov, Ingrid Bemelmans, Laëtitia Jaillardon, Catherine Ibisch, Mario Campone

**Affiliations:** 1 Oniris, Nantes Atlantic College of Veterinary Medicine, Food Science and Engineering, Animal cancers, Models for Research in Comparative Oncology (AMaROC), Site de la Chantrerie, Route de Gachet, CS40706, 44307 Nantes, France; 2 CRCINA, INSERM, Université d’Angers, Université de Nantes, Nantes, France; 3 Department of Pathology, University hospital, Nantes, France; 4 Department of Animal Medicine, Surgery and Pathology, Complutense University of Madrid, Madrid, Spain; 5 Animal and Veterinary Research Centre (CECAV), University of Trás-os-Montes and Alto Douro (UTAD), Vila Real, Portugal; 6 Pathology and Tissue Analytics, Pharma Research & Early Development, Roche Innovation Center Munich, Munich, Germany; 7 Pharmaceutical Sciences, Pharma Research & Early Development, Roche Innovation Center Munich, Munich, Germany; 8 Cerba Vet, Wissous, France; 9 Oniris, Nantes Atlantic College of Veterinary Medicine and Food Sciences, LDHVet, Nantes, France; 10 Institut de Cancérologie de l’Ouest, Angers, France

**Keywords:** Dog, Animal model, Breast cancer, Immunophenotype, Luminal, Triple-negative

## Abstract

**Purpose:**

Relevant animal models of human breast cancer are currently needed, especially for the aggressive triple-negative breast cancer subtype. Recent studies and our results (Part 1) indicate that spontaneous canine invasive mammary carcinomas (CMCs) resemble human breast cancer by clinics and pathology as well as behavior and prognostic indicators. We hypothesized that the current molecular classifications of human breast cancer, used for therapeutic decision, could be relevant to dogs.

**Methods:**

Three hundred and fifty female dogs with spontaneous CMC and a 2-year follow-up were retrospectively included. By immunohistochemistry, CMCs were classified according to Nielsen (Clin Cancer Res 10:5367–5374, 2004) and Blows (PlosOne doi: 10.1371/journal.pmed.1000279, 2010) into the subtypes of human breast cancer.

**Results:**

Four immunophenotypes were defined either according to Nielsen classification (luminal A 14.3%, luminal B 9.4%, triple-negative basal-like 58.6%, and triple-negative nonbasal-like 17.7% CMCs); or to Blows classification (luminal 1−: 11.4%, luminal 1+: 12.3%, Core basal phenotype: 58.6%, and five-negative phenotype: 17.7%). No HER2-overexpressing CMC as defined by a 3 + immunohistochemical score was observed in our cohort. By univariate and multivariate analyses, both immunophenotypical classifications applied to CMCs showed strong prognostic significance: luminal A or luminal 1+ CMCs showed a significantly longer disease-free interval (HR = 0.46), Overall (HR = 0.47), and Specific Survival (HR = 0.56) compared to triple-negative carcinomas, after adjustment for stage.

**Conclusions:**

In our cohort, triple-negative CMCs largely predominated (76%), were much more prevalent than in human beings, and showed an aggressive natural behavior after mastectomy. Dogs are thus potent valuable spontaneous models to test new therapeutic strategies for this particular subtype of breast cancer.

## Introduction

Human breast cancer is a complex disease encompassing different entities with considerable variation in clinical, phenotypical, and molecular attributes [1]. Historically, breast cancer classifications have been based on assessment of histological type and grade [2]. More recently, expression of estrogen receptor alpha (ERα), progesterone receptor (PR) and overexpression of the human epidermal growth factor receptor 2 (HER2) have been included to redefine classification, predict prognosis, and guide therapy in routine clinical practice [3–6]. The roles of these three biomarkers have been reinforced thanks to progress in molecular analysis and understanding of breast cancer biology [7–10]. Studies based on microarray-based gene expression profiling have confirmed and validated the pathogenic role of hormone receptors (luminal tumors) and of the HER2 oncogene (HER2-positive enriched tumors), and the existence of so-called triple-negative breast cancers (TNBCs), which neither express ERα, PR and HER2, nor depend on their oncogenic pathways [7, 11]. The basal-like subtype represents a subset of TNBCs, which expresses genes ordinarily expressed in the basal/myoepithelial cell compartment of normal breast (e.g., cytokeratins CK5, CK6, or CK14) as well as epidermal growth factor receptor (EGFR). The spectrum of triple-negative/basal-like breast cancers is wide but, clinically, most patients have a very poor prognosis with currently no targeted therapy [12].

Gene expression profiling is of limited utility in clinical practice, and immunohistochemical surrogates have been developed. Among luminal tumors (ERα and/or PR positive), the addition of the Ki-67 cell proliferation marker discriminates the luminal A (Ki-67 low) and luminal B (Ki-67 high) subtypes [13]. According to Nielsen et al. the addition of CK5/6 and EGFR helps in identifying basal-like tumors with aggressive features [8]. The panel of these markers has proven useful to predict the risk of recurrence [14]. Using five of these immunohistochemical markers (ERα, PR, HER2, CK5/6 and EGFR), Blows et al. proposed a classification into seven subtypes with prognostic implications: luminal 1+ and luminal 1−, luminal 2+ and luminal 2−, nonluminal HER2-overexpressing, nonluminal core basal phenotype, and nonluminal five-negative phenotype [9]. The prognostic value of both classifications (Nielsen and Blows) has been demonstrated by further studies [15].

Canine invasive mammary carcinomas (CMCs) have been suggested as a valuable spontaneous model of human breast cancer, due to high similarities in terms of epidemiology, pathology, tumor genetics, and biological behavior [16–19]. Immunohistochemical classification of CMCs using the human-based molecular classification has been a recent focus of research [16]. Contradictory results have been obtained due to variable applications of the criteria applied in human breast cancer classification [20–23]. The purpose of this paper was thus to establish the value of the human breast cancer immunohistochemical classification adapted for canine invasive mammary carcinomas in the same large cohort of 350 cases that was used to described the natural history and prognostic factors of CMCs [19]. This study aims thus to contribute to the evaluation of these tumors as potent preclinical models for human breast cancer.

## Methods

### Patients and follow-up

The cohort of canine patients evaluated in this study is described in detail in Part 1 of the present study. Briefly, 350 female dogs with at least one invasive mammary carcinoma, but free from other cancer, were included in this retrospective study. The owners’ written consent and approval from the Oniris College of Veterinary Medicine local Animal Welfare Committee were obtained prior to inclusion. All dogs were treated surgically and none of the dogs received any additional anticancer treatment before and/or after mastectomy. Information on signalment, reproductive history and outcome were obtained from referring veterinarians and owners. All 350 dogs were followed up for at least 48 months in order to study the disease-free interval (DFI, interval from mastectomy to the earliest local recurrence, new primary tumor, lymph node metastasis, and/or distant metastasis), Overall Survival (OS, time between mastectomy and death from any cause), and Specific Survival (SS, time between mastectomy and death attributable to the mammary carcinoma).

### Pathological and immunohistochemical evaluations and classification

Histopathological examination procedures and description of the evaluated criteria were detailed in part 1 [19]. Immunohistochemical (IHC) procedures were described previously [19, 24]. Four veterinary pathologists (JA, FN, LP, and AG) and one medical pathologist (DL) examined the stained slides blindly (i.e., without any information on the dog and without being aware of the results of the other pathologists). In case of discrepancy between evaluators, cases were collectively reviewed in order to achieve a common immunohistochemical score for each parameter.

**Fig. 1:**
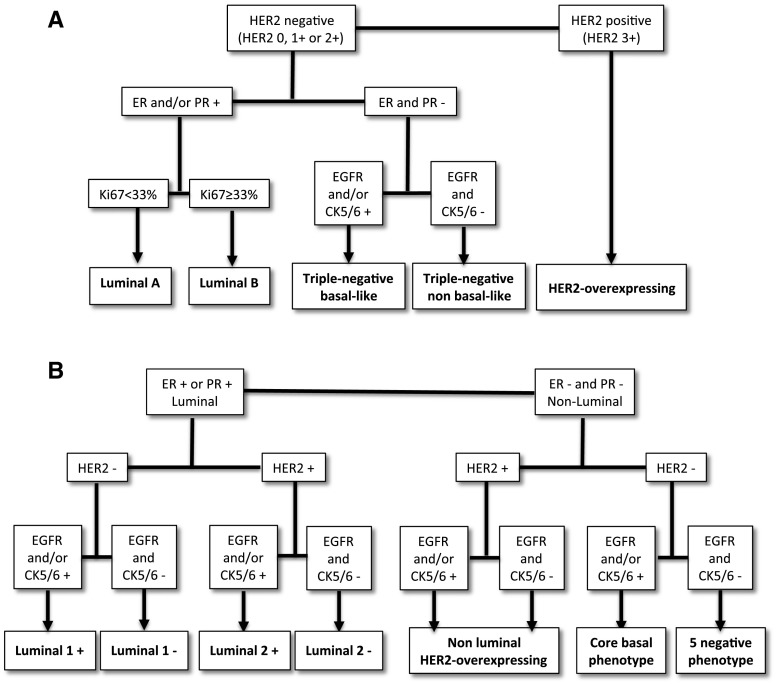
Algorithms of immunohistochemical classifications of canine mammary carcinomas adapted from Nielsen (**A**) and Blows (**Β**)

The combination of 6 immunohistochemical markers (ERα, PR, HER2, Ki-67, CK5/6, and EGFR) was used to define the immunophenotypes of CMCs as defined for human breast cancer according to Nielsen et al. [8] and Blows et al. [9] (Fig. 1A, B).

### Statistical analyses

The MedCalc^®^ statistical software (Ostend, Belgium) was used. Continuous variables are expressed as median [range], mean ± standard deviation. Correlations between categorical variables were analyzed using the Pearson *χ* ^2^ test. The Kaplan–Meier method and logrank tests were used for univariate survival analyses, and Cox proportional hazards models for multivariate survival analyses, whose results are reported using the Hazard Ratio (HR), its confidence interval (95%-CI), and the *p* value of each covariate. For all statistical tests, a *p*-value < 0.05 was considered significant.

## Results

### Relative frequency of CMC immunophenotypes

According to Nielsen (2004), 83 CMCs were classified as luminal (23.7%) including 50 (14.3%) as luminal A and 33 (9.4%) as luminal B. No HER2-overexpressing CMC, as defined by a HER2 score of 3+ , was diagnosed. 267 CMCs were defined as triple-negative CMCs (76.3%) either of the basal-like type (205; 58.6%) or of the nonbasal-like type (62; 17.7%) (Table 1).

**Table 1:** Immunophenotypes of canine invasive mammary carcinomas according to Nielsen [8] and Blows [9] classification

	Number (%)
Immunophenotypes according to Nielsen et al. [8]	
Luminal-A	50 (14.3)
Luminal-B	33 (9.4)
Her2-overexpressing	0 (0)
Triple-negative basal-like	205 (58.6)
Triple-negative nonbasal-like	62 (17.7)
Immunophenotypes according to Blows et al. [9]	
Luminal 1−	40 (11.4)
Luminal 1+	43 (12.3)
Her2-overexpressing	0 (0)
Core basal phenotype	205 (58.6)
5 negative phenotype	62 (17.7)
Total	350 (100)

According to Blows (2010), 83 CMCs were classified as luminal (23.7%), 40 (11.4%) as luminal 1− and 43 (12.3%) as luminal 1+ . As there were no HER2-overexpressing CMCs (HER2 score of 3+), the luminal 2 and non luminal-HER2 subtypes were not observed. Among the 267 triple-negative CMCs (76.3%), the core basal phenotype (205 cases) corresponds to the basal-like subtype of Nielsen classification, and the five-negative phenotype (62 cases) to the nonbasal subtype (Table 1).

### Differences between CMC immunophenotypes

Luminal and triple-negative CMCs, identically defined by Nielsen or Blows, significantly differed by their mean pathologic tumor sizes (*p* = 0.042), by being significantly higher in triple-negative (18 ± 7 mm) than in luminal (16 ± 6 mm) CMCs, and by parameters related to cell proliferation, i.e., mitotic index (*p* = 0.007) and Ki-67 index (*p* = 0.002), being significantly higher in triple-negative CMCs (respectively 43 ± 31 mitoses and 38 ± 17%) compared with luminal CMCs (respectively, 34 ± 21 mitoses and 31 ± 17%).

The CMC immunophenotypes according to Nielsen differed by stage at diagnosis, i.e., regional lymph node status (*p* = 0.036, less commonly pN+ in the luminal A subgroup), and cell proliferation, i.e., mitotic index (*p* = 0.008, higher in triple-negative than in luminal A CMCs) and Ki-67 index (*p* < 0.001), lower in luminal A (19 ± 8%) than in triple-negative CMCs (38 ± 17%), but higher in luminal B (49 ± 11%) than in triple-negative CMCs.

The CMC immunophenotypes according to Blows only differed by cell proliferation, i.e., mitotic index (*p* = 0.015, lower in luminal 1+ CMCs than in five-negative CMCs) and Ki-67 index (*p* = 0.016, lower in luminal 1− CMCs than in core basal CMCs).

### Prognostic factors of luminal CMCs

In the subcohort of 83 dogs with luminal CMC, the distinction between luminal A and B subtypes was a strong prognostic factor by multivariate analyses (Table 2).

**Table 2:** Prognostic factors for dogs with luminal invasive mammary carcinomas by multivariate analysis (*n* = 83)

		HR	95% CI	*p*
Disease-free interval (*p* = 0.0024)				
Multicentricity	Multicentric versus single	3.60	1.20–10.82	0.0234
CK5/6	CK5/6− versus CK5/6+	2.89	1.14–7.36	0.0266
Nielsen immunophenotypes	Lum B versus Lum A	4.00	1.58–10.14	0.0036
Overall survival (*p* = 0.0004)				
Age at diagnosis	≥ 11.7 versus < 11.7 years	2.03	1.22–3.40	0.0069
Blows classification	Lum 1− versus Lum 1+	1.94	1.19–3.18	0.0082
Nielsen immunophenotypes	Lum B versus Lum A	2.10	1.26–3.49	0.0045
Specific survival (*p* = 0.0003)				
Multicentricity	Multicentric versus single	2.81	1.04–7.62	0.0427
Blows classification	Lum 1− versus Lum 1+	2.25	1.11–4.58	0.0254
Nielsen immunophenotypes	Lum B versus Lum A	3.16	1.59–6.28	0.0011

The luminal B phenotype (HR = 4.00), multicentricity (HR = 3.60), and CK5/6 expression (HR = 2.89) were independently associated with disease-free interval (DFI). Shorter overall survival (OS) was associated to the luminal B subgroup (HR = 2.10), the luminal 1− subgroup (HR = 1.94), and older dogs (HR = 2.03). For the risk of cancer-related death (SS), the luminal B (HR = 3.16) and luminal 1− (HR = 2.25) phenotypes were of poor prognosis, independently from multicentricity (HR = 2.81).

To summarize outcome prediction in dogs with luminal CMC, age, multicentricity, Ki-67 index, and basal markers (EGFR and CK5/6) were strong and independent prognostic factors by multivariate analyses.

### Prognostic factors of triple-negative CMCs

In the 267 dogs with triple-negative CMC, Nielsen and Blows classifications were independent prognostic factors by multivariate analyses for DFI and OS but not for SS (Table 3).

**Table 3:** Prognostic factors for dogs with triple-negative invasive mammary carcinomas by multivariate analysis (*n* = 267)

		HR	95% CI	*p*
Disease-free interval (*p* < 0.0001)				
Pathologic nodal stage	pN+ versus pN0-pNX	1.74	1.12–2.72	0.0147
Distant metastasis	M1 versus M0-MX	19.40	6.69–56.27	< 0.0001
Lymphovascular invasion	LVI– versus LVI+	0.38	0.25–0.58	< 0.0001
Nielsen/Blows immunophenotypes	TN non BL (5 neg) versus TNBL (Core phenotype)	1.57	1.03–2.38	0.0359
Overall survival (*p* < 0.0001)				
Pathologic nodal stage	pN+ versus pN0-pNX	1.82	1.33–2.49	0.0002
Peritumoral inflammation	Yes versus no	1.48	1.14–1.93	0.0036
Nielsen/Blows immunophenotypes	TN non BL (5 neg) versus TNBL (Core phenotype)	1.44	1.07–1.94	0.0180
Specific survival (*p* < 0.0001)				
Pathologic tumor size	<20 mm versus ≥ 20 mm	0.66	0.47–0.93	0.0165
Pathologic nodal stage	pN + versus pN0-pNX	1.87	1.29–2.72	0.0010
Distant metastasis	M1 versus M0-MX	2.71	1.07–6.88	0.0373
Peritumoral inflammation	Yes versus No	1.54	1.10–2.16	0.0115
Ki-67	≤ 33.3% versus > 33.3%	0.66	0.47–0.93	0.0184

For DFI, the nonbasal-like (or five-negative) immunophenotype (HR = 1.57) showed higher risk of cancer progression than the basal-like (or core basal) phenotype independently of stage (pathological nodal stage, HR = 1.74, and distant metastasis, HR = 19.40), and lymphovascular invasion (HR = 0.38 when absent). For OS, the nonbasal-like immunophenotype (HR = 1.44) was a poor prognostic indicator independently of the pathological nodal stage (HR = 1.82) and peritumoral inflammation (HR = 1.48). The risk of cancer-related death in dogs with triple-negative CMC was best predicted by tumor stage (pathologic tumor size, pathologic nodal stage, distant metastasis), peritumoral inflammation, and Ki-67 index, than by Nielsen and Blows immunophenotypes.

To summarize outcome prediction in dogs with triple-negative CMC, stage, Ki-67 index, and peritumoral inflammation were strong and independent prognostic factors by multivariate analyses. Basal markers (EGFR, CK5/6) used to distinguish between triple-negative basal-like and nonbasal-like CMCs were of lower prognostic significance in this category of CMC.

### Prognostic significance of CMC immunophenotypic classification

**Fig. 2:**
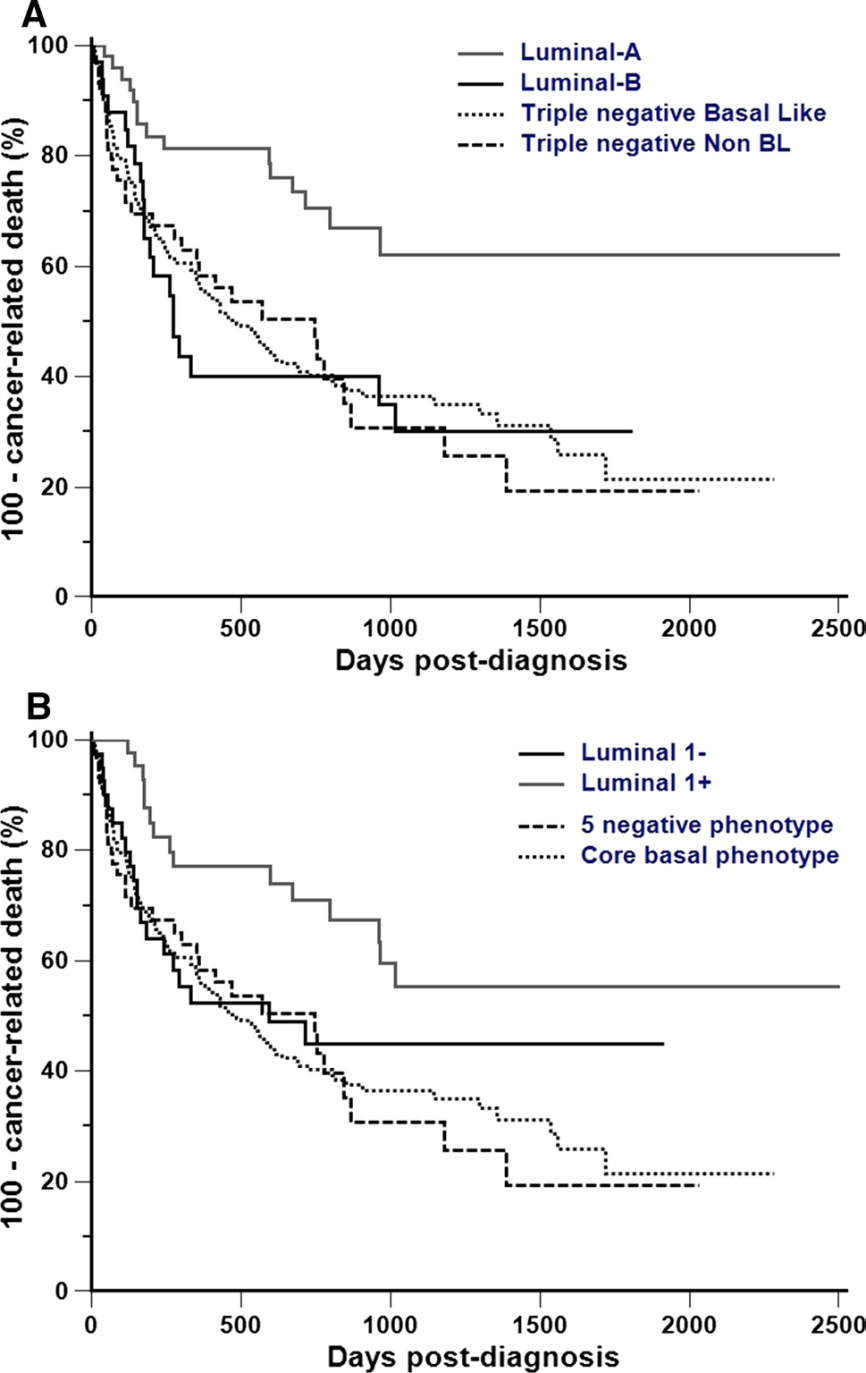
Cancer-specific survival in dogs with mammary carcinoma according to Nielsen (**A**) and Blows (**B**) classifications. **A** According to Nielsen et al. [8], luminal A tumors displayed significantly longer SS (HR = 0.39 [0.23–0.67], Logrank test *p* = 0.0006, Kaplan–Meier curves) than the other immunophenotypes. **B** According to Blows et al. [9], luminal 1+ tumors displayed significantly longer SS (HR = 0.43 [0.25–0.74], Logrank test *p* = 0.0023, Kaplan–Meier curves) than the other immunophenotypes

The natural history of CMCs differed significantly between immunophenotypes. According to Nielsen, luminal A tumors displayed significantly longer DFI (HR = 0.43 [0.24–0.79], *p* = 0.0069), OS (HR = 0.65 [0.46–0.91], *p* = 0.0127), and SS (HR = 0.39 [0.23–0.67], *p* = 0.0006) than the other immunophenotypes (Fig. 2A). According to Blows, luminal 1 + tumors displayed significantly longer DFI (HR = 0.48 [0.26–0.87], *p* = 0.0172), OS (HR = 0.59 [0.40–0.85], *p* = 0.0049), and SS (HR = 0.43 [0.25–0.74], *p* = 0.0023) than the other immunophenotypes (Fig. 2B).

By multivariate analysis, Nielsen classification, pathological nodal stage, and peritumoral inflammation significantly predicted DFI in dogs with CMC (Table 4).

**Table 4:** Prognostic significance of the immunophenotypical classification of canine invasive mammary carcinomas by multivariate analyses (*n* = 350)

		HR	95% CI	*p*
Disease-free survival with Nielsen classification				
Pathologic nodal stage	pN+ versus pN0-pNX	1.99	1.34–2.95	0.0006
Peritumoral inflammation	Yes versus No	1.42	1.01–1.99	0.0464
Nielsen immunophenotypes	Lum A versus TNBL TN non BL versus TNBL	0.46 1.57	0.25–0.85 1.04–2.39	0.0138 0.0339
Disease-free survival with Blows classification				
Pathologic nodal stage	pN+ versus pN0-pNX	2.17	1.48–3.19	0.0001
Margin status	Positive versus negative margins	1.50	1.06–2.11	0.0209
Blows immunophenotypes	Lum 1+ versus Core basal	0.47	0.25–0.85	0.0137
Overall survival with Nielsen classification				
Histological grade	I–II versus III	0.67	0.53–0.86	0.0014
Pathologic nodal stage	pN+ versus pN0-pNX	1.77	1.34–2.34	0.0001
Peritumoral inflammation	Yes versus No	1.41	1.12–1.78	0.0039
Nielsen immunophenotypes	Lum A versus TNBL TN non BL versus TNBL	0.71 1.37	0.50–0.99 1.02–1.85	0.0479 0.0392
Overall survival with Blows classification				
Histological grade	I–II versus III	0.69	0.54–0.87	0.0021
Pathologic nodal stage	pN+ versus pN0-pNX	1.90	1.44–2.49	< 0.0001
Peritumoral inflammation	Yes versus No	1.38	1.10–1.74	0.0061
Blows immunophenotypes	Lum 1+ versus Core basal 5 neg versus Core basal	0.60 1.38	0.41–0.87 1.02–1.86	0.0072 0.0353
Specific survival with Nielsen classification				
Pathologic tumor size	< 20 mm versus ≥ 20 mm	0.73	0.54–0.99	0.0411
Pathologic nodal stage	pN+ versus pN0-pNX	1.87	1.33–2.61	0.0003
Distant metastasis	M1 versus M0-MX	3.15	1.36–7.27	0.0074
Peritumoral inflammation	Yes versus No	1.59	1.18–2.15	0.0027
Nielsen immunophenotypes	Lum A versus TNBL	0.41	0.24–0.70	0.0013
Specific survival with Blows classification				
Pathologic tumor size	<20 mm versus ≥ 20 mm	0.73	0.54–0.99	0.0464
Pathologic nodal stage	pN+ versus pNX	1.94	1.39–2.70	0.0001
	pN0 versus pNX	0.55	0.32–0.96	0.0355
Distant metastasis	M1 versus M0-MX	2.41	1.04–5.60	0.0418
Peritumoral inflammation	Yes versus No	1.50	1.11–2.02	0.0082
Blows immunophenotypes	Lum 1+ versus Core basal	0.56	0.36–0.86	0.0092

The strongest prognostic factor was nodal metastasis (HR = 1.99), followed by Nielsen classification with luminal A (HR = 0.46) and triple-negative nonbasal-like tumors (HR = 1.57) significantly differing from triple-negative basal-like CMCs. For DFI, Blows classification, nodal metastasis, and positive margins were independent prognostic factors by multivariate analysis (Table 4), with luminal 1+ CMCs (HR = 0.47) of significantly lower risk of cancer progression compared with core basal triple-negative CMCs.

OS was predicted by 4 independent parameters: Nielsen or Blows classification, histological grade, pathological nodal stage, and peritumoral inflammation (Table 4). The luminal A and luminal 1+ immunophenotypes have favorable prognoses, whereas the nonbasal-like phenotype was associated with shorter OS. Thus, the dogs with lowest mortality rate following diagnosis of CMC, were those with a smaller (< 20 mm) grade I–II carcinoma, without nodal metastasis, without significant peritumoral inflammation, and of the luminal A (Nielsen) or luminal 1+ (Blows) immunophenotype.

Nielsen and Blows classifications were also strong prognostic parameters for SS, by multivariate analysis, together with pathologic tumor size, pathological nodal stage, distant metastasis, and peritumoral inflammation (Table 4). Luminal A (HR = 0.41) and luminal 1+ (HR = 0.56) CMCs were of better prognosis than triple-negative basal-like CMCs. These results highlight the strong prognostic influence of peritumoral inflammation in CMCs, independently from the immunophenotypes and stage at diagnosis.

In conclusion, both Nielsen and Blows immunophenotypic classifications could be applied to canine mammary carcinomas and defined tumor subgroups of distinctive clinicopathological features and outcomes. Both classifications were strong and independent prognostic factors for CMCs.

## Discussion

The spontaneous occurrence of canine mammary tumors has long been claimed to provide a suitable model for human breast cancer [16, 25, 26]. Until recently, however, the classification of canine mammary tumors did not consider the prognostic elements that are contemplated in the classification of human breast cancer: the concurrent expression of the pivotal cancer-related biomarkers such as ERα, PR, HER2, Ki-67, and basal markers have thus been evaluated only in a very few studies, dealing either with small cohorts or without complete follow-up [17, 22, 27]. Due to the critical value of these parameters in human breast cancer assessment for prognosis and therapeutic guidance, the validation of spontaneous CMCs as models for human pathology and preclinical assays required the use of comparable evaluation and classification criteria [28].

The present study applied, in the largest CMC cohort reported to date (350 female dogs), the antibodies used to characterize the molecular groups in routine human pathology, according to Nielsen et al. [8], Cheang et al. [13], and Blows et al. [9]. We identified in our cohort, 4 of the 5 subtypes defined by Nielsen in human breast cancer. In dogs, a low rate of luminal tumors was observed (total of 23.7%), and no HER2-overexpressing tumors were found (defined by a score of 3 + by HER2 immunohistochemistry). A vast majority of CMCs were of the triple-negative subtype (58.6% triple-negative basal-like or core basal phenotype; and 17.7% triple-negative nonbasal-like, or five-negative phenotype; total of 76.3%), associated with a shorter survival, as reported in human breast cancer [12, 29].

The proportion of the different subtypes in our cohort of 350 female dogs differed significantly from the few papers reporting the application of human breast cancer immunohistochemical classification to dogs [21, 23, 27]. Several reasons may explain these discrepancies, including variable inclusion criteria and methodological aspects. In those previous studies, where the invasive nature of the CMC has not been consistently confirmed by p63 immunohistochemistry, the higher incidence of carcinomas in situ may explain the high level of hormone receptor-positive (luminal) neoplasms. Furthermore, assessment of marker expression (i.e., antibodies clones and thresholds for positivity) was variable and often distinct from those recommended for human breast cancer [3]. In our study, consensus diagnoses on the immunohistochemical interpretation involving five veterinary and medical pathologists have been achieved in a comparative pathology perspective.

In the 83 luminal CMCs reported here, patient age, multicentricity, Ki-67 index (used to distinguish between luminal A and luminal B tumors), and basal marker expression (EGFR and CK5/6, used to differentiate luminal 1− from luminal 1+ tumors) were strong and independent prognostic factors. Although the prognostic value of the proliferation index has been recognized for a long time in human breast cancer [13, 30–32] as in canine mammary carcinomas [33], the heterogeneity of luminal breast cancer in terms of immunohistochemical expression of basal markers is currently an active area of research [34]. Such investigations remain to be done in CMCs.

In the 267 triple-negative CMCs, similarly defined by Nielsen and Blows classifications, the stage (pathological tumor size, nodal stage, and distant metastasis), Ki-67 index, and peritumoral inflammation were strong and independent prognostic factors by multivariate analyses, but the expression of basal markers (EGFR and CK5/6) was not. In human breast cancer, contradictory results have been reported about the prognostic significance of basal marker expression [35, 36]. Compared with grade-matched nonbasal-like cancers, carcinomas with a basal-like phenotype were not associated with a poorer outcome in some studies, whereas a more adverse prognosis was observed in others [36–38]. Interestingly, the independent prognostic value of peritumoral inflammation observed in the triple-negative subtype of this canine cohort could reflect the importance of immune reaction in canine mammary carcinomas, similar to what was described in recent gene expression studies of TNBCs, which identify distinct subtypes based on immune activation and immune suppression [39, 40]. Further evaluation of the precise composition of the inflammatory infiltrate (e.g., CD8+ lymphocytes, regulatory T lymphocytes, M1 or M2 subsets of macrophages) is required to define the significance of the immune microenvironment in canine carcinomas [41–43].

In our study, the CMC immunophenotypes according to Nielsen differed from each other by stage at diagnosis and cell proliferation, and the CMC immunophenotypes according to Blows also differed from each other by cell proliferation. Thus, luminal and triple-negative CMCs displayed significant distinctive pathological features indicative of intrinsic distinct biological characteristics, such as pathologic tumor size and proliferative activity (as defined by mitotic index and Ki-67 index). Similar differences have been reported in human breast cancers [44]. However, other features that were described as distinctive between luminal and triple-negative breast carcinomas, such as reproductive history, histological grade, and basal marker expression [15], did not significantly differ between luminal and triple-negative carcinomas in dogs.

Interestingly, the immunophenotypic classification is also of prognostic significance in dogs, with luminal A and luminal 1− CMCs displaying a significantly longer Disease-Free Interval (HR = 0.46), Overall Survival (HR = 0.47, and Specific Survival (HR = 0.56) compared with triple-negative carcinomas, after adjustment for stage. This positive influence of hormone receptor expression on prognosis has been reported in dogs [45, 46] as in women [13, 47, 48].

In our cohort, no HER2-overexpressing tumors as defined by a 3+ immunohistochemical score were observed. Previous studies dealing with canine mammary tumors have, however, reported significant levels of HER2 expression in CMC without any agreement on its prognostic value [27, 49, 50]. Nevertheless, the existence of HER2-overexpressing mammary tumors in dogs has to be considered as doubtful for methodological reasons (selection of antibodies, dilution, scoring criteria, and absence of appropriate controls). In our study, the immunohistochemical protocols and criteria used in human breast cancer evaluation [51] have been used, with adequate internal and external controls, and evaluation by a pathologist experienced in human breast cancer pathology. No 3 + score suggestive of HER2 gene amplification and protein overexpression has been detected in our canine population. This result is in agreement with a recent study using antibody-based, transcriptomic and mass spectrometry analysis of HER2 expression in canine mammary tumors. In this study, the immunohistochemical results suggested a lack of specificity of the FDA-approved antibody A0485 in canine samples, as further demonstrated by Western immunoblotting and reverse-phase protein arrays. Furthermore, HER2 was not detected by mass spectrometry in an immunohistochemically positive carcinoma [52]. These results, like the ones of the present study, are in favor of the absence or at least the high rarity of HER2-overexpressing mammary carcinomas in dogs, but need to be confirmed by molecular methods, either in situ at the gene level (such as specific in situ hybridization) or by RNA expression in a large cohort.

## Conclusions

In conclusion, the immunohistochemical classification of human breast cancer, commonly used to characterize the molecular groups in human pathology, may be applied to canine mammary carcinomas. CMCs thus appear as a heterogeneous group of distinct molecular-driven tumors, like human breast cancer. Our results must, however, be confirmed in a large gene expression profile molecular study, as only few and preliminary works have been published to date for CMC molecular characterization [53–55].

In our cohort of 350 dogs, triple-negative mammary carcinomas largely predominated (76%) and were much more prevalent than in human beings. They behaved, however, similarly, with a naturally aggressive course after mastectomy. Dogs are thus potent valuable spontaneous cancer models to test new therapeutic strategies, particularly for human triple-negative breast cancer.

## Abbreviations

5 neg: 5 negative
95%-CI: 95% confidence interval
BL: Basal-like
CK: Cytokeratins
CMC: Canine mammary carcinoma
DFI: Disease-free interval
EGFR: Epidermal growth factor receptor (type 1)
ERα: Estrogen receptor alpha
FDA: Food and Drug Administration
HER2: Human epidermal growth factor receptor type 2
HES: Hematoxylin–eosin–saffron
HR: Hazard ratio
IHC: Immunohistochemistry
Lum: Luminal
LVI: Lymphovascular invasion
M: Distant metastasis
OS: Overall survival
PR: Progesterone receptor
pT: Pathologic tumor size
pN: Pathologic nodal stage
RNA: Ribonucleic acid
SS: Specific survival
TN: Triple-negative
TNBCs: Triple-negative breast cancers
